# Evaluation of stability of stereotactic space defined by cone‐beam CT for the Leksell Gamma Knife Icon

**DOI:** 10.1002/acm2.12073

**Published:** 2017-04-17

**Authors:** Ismail AlDahlawi, Dheerendra Prasad, Matthew B. Podgorsak

**Affiliations:** ^1^ Department of Radiation Medicine Roswell Park Cancer Institute Buffalo NY USA; ^2^ Department of Neurosurgery Roswell Park Cancer Institute Buffalo NY USA; ^3^ Department of Physiology and Biophysics State University of New York Buffalo NY USA; ^4^ Department of Neurosurgery Jacobs School of Medicine and Biomedical Sciences State University of New York Buffalo NY USA; ^5^ Department of Radiation Oncology King Fahad Specialist Hospital‐Dammam Dammam Saudi Arabia

**Keywords:** CBCT, Gamma knife, icon, Leksell space, QA tool, stereotactic radiosurgery

## Abstract

The Gamma Knife Icon comes with an integrated cone‐beam CT (CBCT) for image‐guided stereotactic treatment deliveries. The CBCT can be used for defining the Leksell stereotactic space using imaging without the need for the traditional invasive frame system, and this allows also for frameless thermoplastic mask stereotactic treatments (single or fractionated) with the Gamma Knife unit. In this study, we used an in‐house built marker tool to evaluate the stability of the CBCT‐based stereotactic space and its agreement with the standard frame‐based stereotactic space. We imaged the tool with a CT indicator box using our CT‐simulator at the beginning, middle, and end of the study period (6 weeks) for determining the frame‐based stereotactic space. The tool was also scanned with the Icon's CBCT on a daily basis throughout the study period, and the CBCT images were used for determining the CBCT‐based stereotactic space. The coordinates of each marker were determined in each CT and CBCT scan using the Leksell GammaPlan treatment planning software. The magnitudes of vector difference between the means of each marker in frame‐based and CBCT‐based stereotactic space ranged from 0.21 to 0.33 mm, indicating good agreement of CBCT‐based and frame‐based stereotactic space definition. Scanning 4‐month later showed good prolonged stability of the CBCT‐based stereotactic space definition.

## 1 Introduction

Stereotactic radiosurgery (SRS) delivers a high dose of radiation to a target while sparing healthy structures, and this mandates precise localization. Traditionally, Gamma Knife SRS treats intracranial lesions and involves localizing the target coordinates based on an invasive frame fixed to the patient skull.^1^ With the advances in image‐guided radiotherapy, the possibility of localizing targets using images allows for noninvasive frameless stereotactic radiosurgery, as well as for fractionated stereotactic radiotherapy. The new Gamma Knife model, Leksell Gamma Knife^**®**^ Icon^**™**^, has been recently introduced and includes a cone‐beam CT (CBCT) which can be used to define the 3D stereotactic coordinate space without the need for an invasive frame system. The CBCT can be used to define the stereotactic space coordinates for either G‐frame treatments or the new frameless thermoplastic mask system. This study focused on the stability of the CBCT‐based stereotactic space definition and its agreement with the standard frame‐based stereotactic space definition, as one element in the overall delivery accuracy chain of the Gamma Knife Icon. Other studies have looked at the accuracy of Gamma Knife delivery when using a thermoplastic mask system for skull immobilization with an IR camera and CBCT;^2^ and at quantifying translational and rotational shifts when using the invasive frame on a prototype CBCT image‐guided Gamma Knife Perfexion unit.^3^

The purpose of this work was to evaluate the stability of the CBCT‐based stereotactic coordinate space and confirm it is in agreement with the standard frame‐based stereotactic coordinate system throughout a partial volume of the defined stereotactic space.

## 2 Materials and methods

### 2.A Leksell Gamma Knife^®^ Icon^™^ system

Leksell Gamma Knife^**®**^ systems have been designed by the manufacturer (Elekta Instruments, A.B., Stockholm, Sweden) to precisely deliver stereotactic treatments to intracranial targets. The latest design, the Leksell Gamma Knife^**®**^ Icon^**™**^, is identical in the core radiation unit to its predecessor, the Leksell Gamma Knife^**®**^ Perfexion^**™**^ (i.e., 192 Co‐60 sources distributed over eight sectors that can be moved independently to deliver an isocenteric treatment). The new model Icon, however, comes with a cone‐beam CT (CBCT) system for image guidance and a couch‐mounted infrared camera for intrafraction motion management, allowing for frameless thermoplastic mask‐based stereotactic radiosurgery and fractionated stereotactic radiotherapy treatments. The CBCT system is composed of a rotating anode X‐ray tube (RTM 75H, Industria Applicazioni Elettroniche, Cormano MI, Italy) and a 34 cm × 39 cm flat‐panel x‐ray detector (Pixium CBCT 2630, Thales Electron Devices SAS, France) mounted on an arm allowing a 210 degree rotation for scanning. CBCT can be used for obtaining a reference image and determining the Leksell stereotactic space coordinates (hereafter called CBCT‐based stereotactic coordinates), and also can be used prior to treatment delivery for verifying the actual skull position and determining translational and rotation shifts based on coregistration with the reference CBCT image^4^ so that the shot positions are adapted to the target and plan dose distribution is recalculated.^5^ The intrafraction motion management system (IFMM) is composed of an infrared camera (Polaris Vicra, Northern Digital Inc., Waterloo, ON, Canada) mounted on the far end of the patient couch and is used to monitor the movement of a reflector marker placed on the patient nose tip when using thermoplastic mask for immobilization, with the option of automatically stopping the delivery if the movement exceeds a threshold that can be set from 0.5 mm up to 3 mm.^6^, ^7^

### 2.B CBCT stereotactic space definition and CBCT precision QA

**Figure 1:**
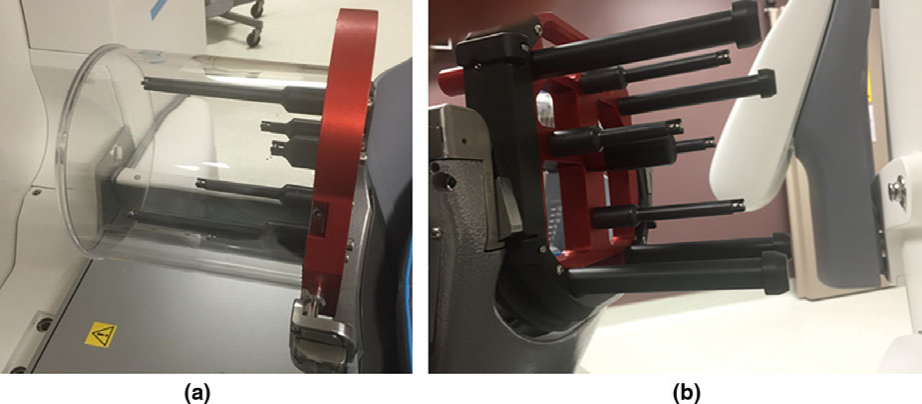
(a) The manufacturer's CBCT tool used for calibrating the CBCT position, and (b) the QA tool Plus used in daily CBCT precision tests.

A special calibration tool is used by the manufacturer's service engineer to find the CBCT to Leksell coordinate transform between the uncalibrated CBCT image and the radiation delivery unit (Fig. 1(a)). The tool consists of six steel ball‐bearings with known Leksell coordinates. An algorithm running in service mode uses the projection images of a CBCT scan of this special tool to calculate the transform between CBCT image and the Leksell coordinates systems.^8^ This calibration procedure was performed once by the manufacturer's service engineer at the time of treatment unit commissioning.

The manufacturer also provides a user QA tool (QA tool Plus) to enable each user to test the CBCT precision (Fig. 1(b)). This CBCT precision QA test is performed daily as part of a comprehensive QA program. The QA tool Plus has four posts each with a steel ball‐bearing. The four steel ball‐bearings are distributed such that they only have three distinguished coordinates in the lateral direction (*X*), three distinguished coordinates in the vertical direction (*Y*), and two distinguished coordinates in the longitudinal direction (*Z*). The preprogrammed CBCT precision test algorithm finds the location of the four ball‐bearings from a CBCT scan of this tool and compares them with their baseline coordinates given during the calibration of the tool by the service engineer.^6^, ^9^ The algorithm calculates each fiducial deviation as well as the maximum deviation of tests points in the reconstructed CBCT image volume, and the test is considered “passed” if the maximum deviation in image volume is within the acceptable limits (< 0.4 mm). A limitation of the QA tool Plus is that it cannot be attached to the standard CT fiducial indicator box to be CT‐scanned and determine the traditional frame‐based Leksell coordinates, making it problematic for the user to independently verify the validity of Leksell coordinates based on CBCT.

### 2.C In‐house marker tool

**Figure 2:**
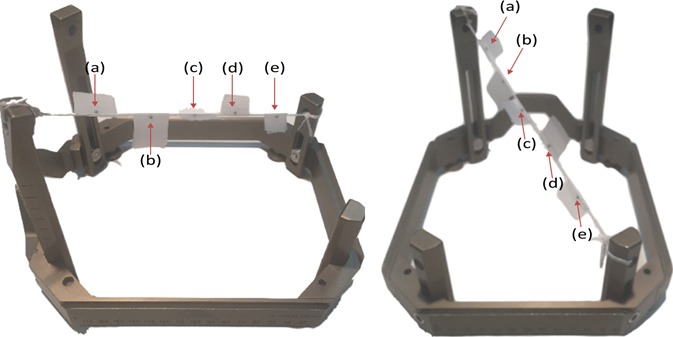
Different views of the in‐house marker tool: an assembled G stereotactic frame with five ball‐bearing markers (pointed with red arrows) attached to a taut string tied to two posts of the G‐frame.

**Figure 3:**
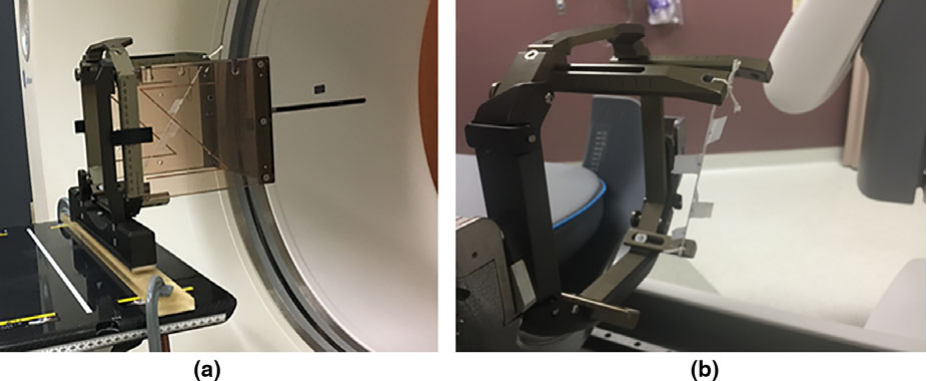
(a) CT scanning of the in‐house marker tool with the CT indicator box attached to; and (b) the same tool mounted on the Gamma Knife Icon couch adapter in preparation for CBCT.

A simple tool with fixed fiducial markers that can easily be localized in both CT and CBCT scans was designed and implemented, similar to the tool used in a previous work,^10^ to independently test the stability and accuracy of the CBCT‐based stereotactic space coordinates. The tool consists of a taut string that is attached to an assembled stereotactic frame (Leksell model G, Elekta, Atlanta, GA, USA), drawn from the left anterior post to the right posterior post. Five ball‐bearing fiducial markers (0.5 mm diameter) were rigidly attached to the string, spanning a broad portion of the stereotactic space (70 mm × 120 mm × 55 mm) and distributed such that each of the five markers gives a unique lateral (*X*), vertical (*Y*), and longitudinal (*Z*) coordinates (Fig. 2). For the reference frame‐based stereotactic coordinate definition, CT scans of this tool attached with the standard CT fiducial indicator box (Fig. 3(a)) were acquired using a CT‐simulator (LightSpeed RT16, GE, San Diego, CA, USA) in an axial mode with a 0.625 mm slice thickness at 120 kV and 350 mA. The CT fiducial box was aligned with a set of CT‐simulation lasers that are well‐maintained in our quality assurance program. The CT images were imported to the Leksell GammaPlan (LGP) software V11.0.2, where the frame‐based Leksell coordinates were determined for each marker. The CT scans were acquired at the beginning, midway, and at end of this study period (6 weeks) to confirm the stability of our tool. For the CBCT‐based stereotactic coordinate definition, CBCT images of the tool (Fig. 3(b)) were taken every working day for 6 weeks using the two predefined scanning settings: CTDI 6.3 mGy (high quality) preset at 90 kV and 25 mA; and CTDI 2.5 mGy (low dose) preset at 90 kV and 10 mA. The CBCT images were automatically imported to the LGP software upon scanning. The pixel sizes for both the CT and CBCT images were 0.5 mm (*X*) by 0.5 mm (*Y*). However, when projecting these images in the LGP software, the pixel sizes are interpolated and became 0.1 mm (*X*) by 0.1 mm (*Y*).

**Figure 4:**
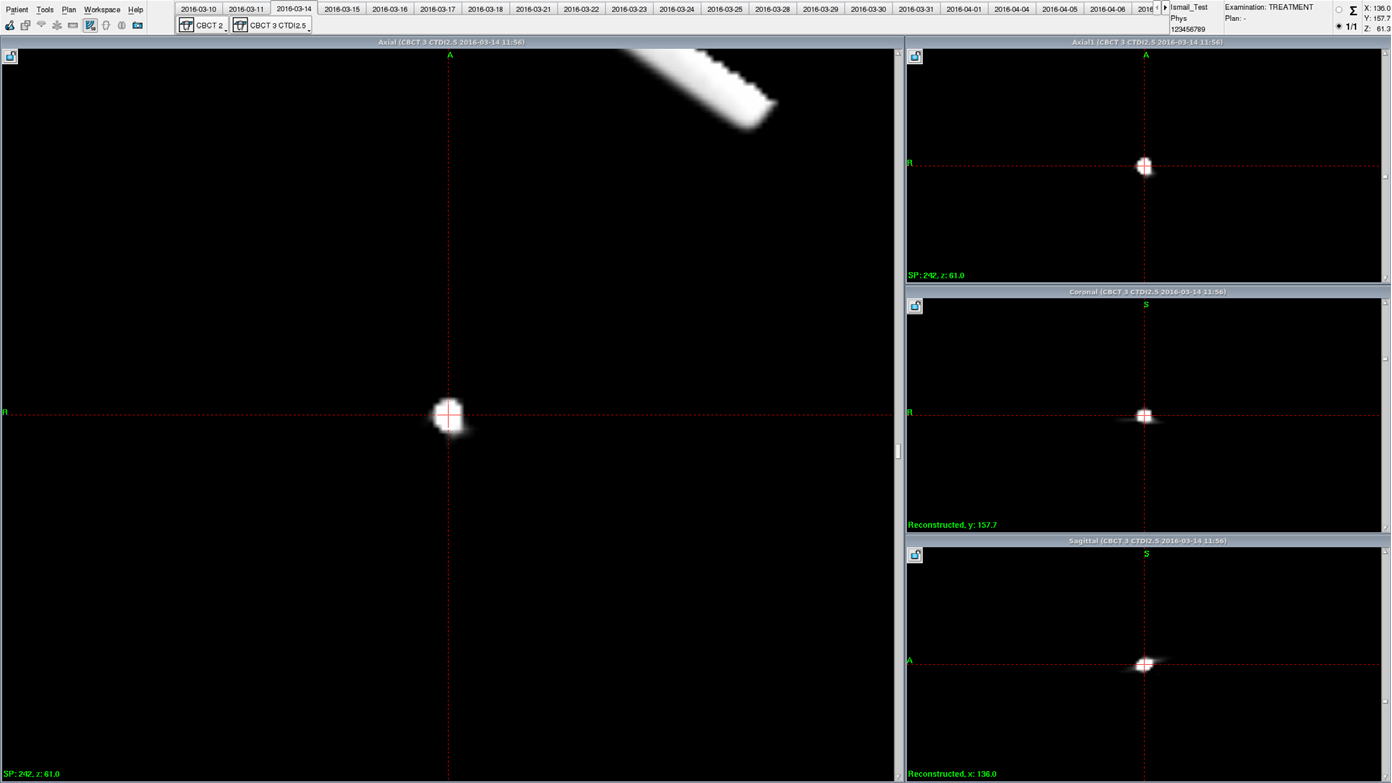
A snapshot of the Leksell GammaPlan treatment planning software showing a zoomed‐in and maximally contrasted CBCT image of one of the markers (marker (a) is shown here). The coordinates reported for each marker were the average of three readings of the geometrical center of markers as determined visually by a single observer.

The LGP software was used to determine the (*X, Y, Z*) and (*X′, Y′, Z′*) coordinates of each marker for each image set of the CT and CBCT scans, respectively. The geometric center of each marker was determined by a single observer looking for the center of each “fuzzy” enhancement in maximum zoomed‐in and maximum contrasted images, as shown in Fig. 4. To examine the reproducibility of determining the marker center, the coordinates of each marker were read three times in separate instances by the observer, and the average of the three readings was reported. The magnitude of vector difference (*r*) between the mean coordinates of frame‐based and CBCT‐based was calculated as:

$$r=\sqrt{{\left(\overset{¯}{X}-{\overset{¯}{X}}^{\prime }\right)}^{2}+{\left(\overset{¯}{Y}-{\overset{¯}{Y}}^{\prime }\right)}^{2}+{\left(\overset{¯}{Z}-{\overset{¯}{Z}}^{\prime }\right)}^{2}}.$$

Furthermore, an additional CBCT of the markers frame tool was scanned 4 months later to determine the long‐term stability of the CBCT‐based stereotactic coordinate system.

## 3 Results and discussion

**Figure 5:**
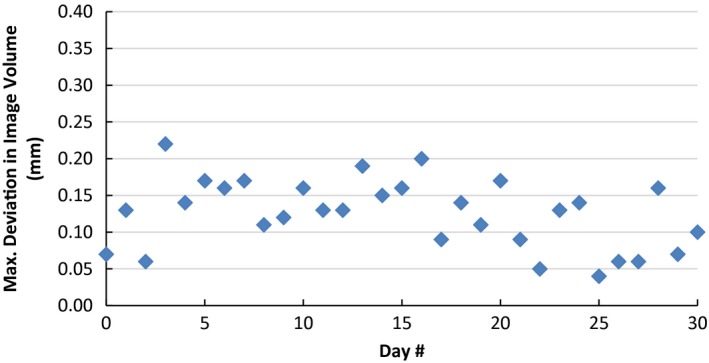
Results of daily CBCT precision QA test. The graph shows the variation of maximum deviation in image volume between the daily QA tool Plus and the expected baseline taken during calibration of the tool.

The daily CBCT precision QA test using the manufacturer's QA tool Plus resulted in a mean of 0.13 mm maximum deviation in image volume between the daily tests and the calibration, with a standard deviation of 0.05 mm and a maximum of 0.22 mm. These daily tests met the manufacturer's limit of 0.4 mm in maximum deviation in image volume, indicating a good reproducibility in CBCT coordinate positions, as shown in Fig. 5.

The reproducibility in determining center of the markers within the frame tool can be quantified as the mean of the standard deviations of three readings of markers coordinates in each CT/CBCT scan set. This was found to be approximately 0.05 mm, with a maximum of 0.13 mm.

There was no statistical significant difference (*P*‐value > 0.05 using t‐test) in determining the marker positions in CBCT images taken using the two different scanning preset settings (CTDI 6.3 mGy vs CTDI 2.5 mGy). This is expected in our case as the ball‐bearings are easily identified in either scanning preset because of their high contrast with the surrounding air, and as the CBCT to Leksell coordinates calibration is independent of scanning preset used.

**Figure 6:**
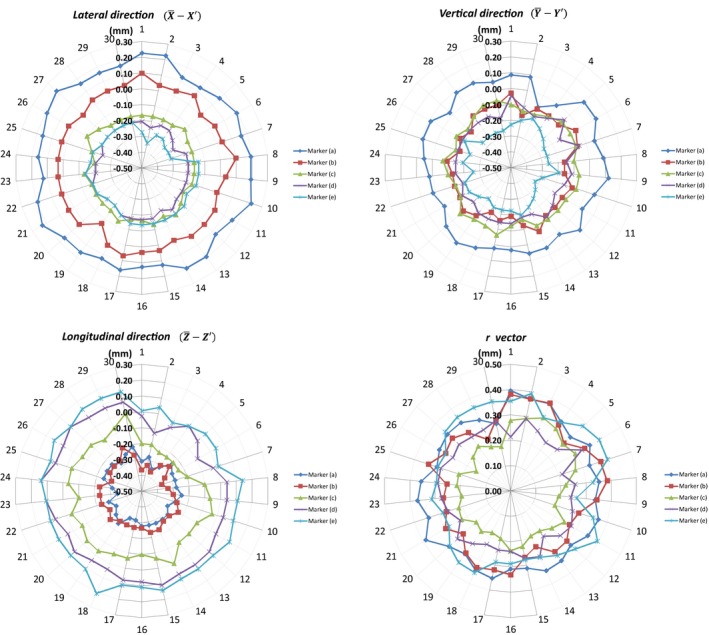
Daily variations of CBCT‐based stereotactic coordinates from the mean frame‐based in the lateral, vertical, and longitudinal directions and the magnitude of vector difference (*r)* of each marker over a period of 6 weeks.

The daily variations in the difference between frame‐based and CBCT‐based of each of the five markers positions in the lateral, vertical, and longitudinal directions and the *r* vector difference magnitudes are shown in Fig. 6. The graphs indicate good stability of the CBCT‐based coordinates over the 6‐week period, with standard deviations being less than 0.07 mm for markers positions. The maximum deviation in the magnitude of vector difference (*r*) of the CBCT‐based from the frame‐based stereotactic definition during this period was noted to be 0.40 mm, at the end edges of the stereotactic frame [i.e., marker (a) & (e)].

The magnitude of vector difference (*r*) between mean coordinates of the frame‐based and the CBCT‐based for the five markers ranged from 0.21 to 0.31 mm (Table 1). Small shift trends in the magnitude and direction of the frame‐ and CBCT‐based coordinate mean differences were noted as we go from one edge of the stereotactic space to another; i.e., from marker (a) at the most left‐anterior‐superior point to marker (e) at the most right‐posterior‐inferior point. In the lateral direction, the mean difference between frame‐ and CBCT‐based coordinates (i.e., $\left(\overset{¯}{X}-{\overset{¯}{X}}^{\prime }\right)$) ranged from 0.17 mm for marker (a) to −0.20 mm for marker (e), with the positive sign indicating the frame‐based coordinate being larger than CBCT‐based coordinate value; in the vertical direction, the mean difference $\left(\overset{¯}{Y}-{\overset{¯}{Y}}^{\prime }\right)$ ranged from 0.05 mm for marker (a) to −0.23 mm for marker (e); and in the longitudinal direction, the mean difference $\left(\overset{¯}{Z}-{\overset{¯}{Z}}^{\prime }\right)$ ranged from −0.28 mm for marker (a) to 0.10 mm for marker (e). The data suggest there is a small systematic difference between the frame‐based and CBCT‐based stereotactic spaces in our particular unit. The data also suggest that the Leksell space as determined by the CBCT in our unit is “compressed” compared to the frame‐based space, i.e., by about 0.5% in the X‐direction (69.2 mm in the CBCT case vs 69.57 mm in the frame case), 0.3% in the Y‐direction (120.62 vs 120.92), and by 0.7% in the Z‐direction (53.87 vs 54.26).

**Table 1:** Mean ± standard deviation and range of coordinates of each marker in frame‐based stereotactic space (*X*,* Y*,* Z*) and CBCT‐based stereotactic space (*X*′*, Y*′, *Z*′). The standard deviation is calculated over all daily readings taken. The magnitude of vector difference *r* is calculated as $r=\sqrt{{\left(\overset{¯}{X}-{\overset{¯}{X}}^{\prime }\right)}^{2}+{\left(\overset{¯}{Y}-{\overset{¯}{Y}}^{\prime }\right)}^{2}+{\left(\overset{¯}{Z}-{\overset{¯}{Z}}^{\prime }\right)}^{2}}$

Marker ID	Frame‐based coordinates (mm)			CBCT‐based coordinates (mm)			$\overset{¯}{r}$ (mm)
	$\overset{¯}{X}$	$\overset{¯}{Y}$	$\overset{¯}{Z}$	${\overset{¯}{X}}^{\prime }$	${\overset{¯}{Y}}^{\prime }$	${\overset{¯}{Z}}^{\prime }$	
a	136.23 ± 0.20 (135.97–136.43)	157.69 ± 0.20 (157.43–158.00)	61.05 ± 0.22 (60.87–61.43)	136.06 ± 0.03 (136.00–136.10)	157.63 ± 0.04 (157.57–157.77)	61.34 ± 0.03 (61.27–61.40)	0.33 ± 0.21
b	121.23 ± 0.25 (120.93–121.57)	128.41 ± 0.12 (128.27–128.57)	75.63 ± 0.22 (75.30–75.90)	121.20 ± 0.03 (121.13–121.30)	128.52 ± 0.04 (128.43–128.60)	75.91 ± 0.04 (75.83–76.00)	0.30 ± 0.21
c	105.93 ± 0.22 (105.77–106.30)	101.43 ± 0.11 (101.27–101.53)	85.40 ± 0.11 (85.30–85.57)	106.09 ± 0.02 (106.03–106.13)	101.53 ± 0.03 (101.50–101.60)	85.50 ± 0.07 (85.40–85.63)	0.21 ± 0.18
d	85.99 ± 0.24 (85.73–86.40)	71.85 ± 0.11 (71.67–71.93)	97.31 ± 0.12 (97.20–97.47)	86.20 ± 0.02 (86.17–86.27)	71.99 ± 0.04 (71.90–72.03)	97.26 ± 0.06 (97.17–97.43)	0.25 ± 0.21
e	66.66 ± 0.27 (66.37–67.03)	36.77 ± 0.10 (36.63–36.83)	115.31 ± 0.15 (115.13–115.57)	66.86 ± 0.06 (66.80–67.00)	37.01 ± 0.04 (36.93–37.10)	115.21 ± 0.05 (115.10–115.33)	0.32 ± 0.19

Though the differences are small in our explored stereotactic volume (limited to 70 mm × 120 mm × 55 mm), the trend might indicate a larger discrepancy in the whole stereotactic space. Unit‐specific assessments, using an independent tool similar to what we described in this study, can be used in assisting decision‐making of selecting which stereotactic reference (i.e., CBCT‐based vs. frame‐based) to be used for determining the Leksell coordinates depending on the accuracy required for each clinical case. Johansson et al. evaluated the geometric accuracy of the Gamma Knife Icon with an end‐to‐end phantom test case and measured the error to be < 0.2 mm, and concluded that the CBCT system of the Icon can accurately be used for patient positioning.^11^

The in‐house markers tool was also scanned 4 months later to determine the prolonged stability of CBCT‐based stereotactic definition. The coordinates of the five markers were within the range of measurements performed in the initial 6‐week study period, indicating good CBCT‐based coordinate definition prolonged stability.

## 4 Conclusion

A simple in‐house tool was used to test the stability of the CBCT‐defined stereotactic space in Gamma Knife Icon and its agreement with the standard frame‐defined stereotactic space, independently from the manufacturer provided tool and methodology. CBCT‐based stereotactic space definition in Gamma Knife Icon was found to be stable over a period of 4 months, and in good agreement with the standard frame‐based stereotactic space definition.

## Conflict of interest

The authors have no conflicts of interest to disclose.

## References

[1] Leksell L . The stereotaxic method and radiosurgery of the brain. Acta Chir Scand. 1951 Dec ;102(4):316–319. 14914373

[2] Li W , Bootsma G , Von Schultz O , et al. Preliminary evaluation of a novel thermoplastic mask system with intra‐fraction motion monitoring for future use with image‐guided Gamma knife. Cureus. 2016;8:e531. 27081592 10.7759/cureus.531 PMC4829406

[3] Li W , Cho Y‐B , Ansell S , et al. The use of cone‐beam computed tomography for image‐guided Gamma knife stereotactic radiosurgery: initial clinical evaluation. Int J Radiat Oncol. 2016;96(1):214–220. 10.1016/j.ijrobp.2016.04.011 27511857

[4] Eriksson M , Nordström H . Design and performance characteristics of a cone Beam CT system for the LGK perfexion In: 17th International Leksell Gamma Knife Society Meeting. New York: Leksell Gamma Knife Society; 2014.

[5] Elekta Instrument AB . Automatic positional delivery correction using a stereotactic CBCT in Leksell Gamma Knife^®^ Icon^™^ (White Paper), 2015. Art. No. 1518415.01.

[6] Elekta Instrument AB . Leksell Gamma knife^®^ Icon^™^ instructions for use (Doc. ID: 1505194 Rev. 01). Stockholm, Sweden; 2015.

[7] Elekta Instrument AB . High definition motion management ‐ enabling stereotactic Gamma Knife^®^ radiosurgery with non‐rigid patient fixations (White Paper); 2015. Art. No. 1509395.03.

[8] Elekta Instrument AB . Position accuracy analysis of the stereotactic reference defined by the CBCT on Leksell Gamma Knife^®^ Icon^™^ (White Paper); 2015. Art. No. 1509392.03.

[9] Elekta Instrument AB . Geometric quality assurance for Leksell Gamma knife^®^ Icon^™^ (White Paper); 2015. Art. No. 1518416.01.

[10] Cernica G , Wang Z , Malhotra H , de Boer S , Podgorsak MB . Investigation of Gamma Knife image registration errors resulting from misalignment between the patient and the imaging axis. Med Phys. 2006;33(4):941. 16696470 10.1118/1.2179751

[11] Johansson J , Nordström H , Eriksson M . Evaluation of the geometric accuracy of the Leksell Gamma knife^®^ Icon^™^ In: 18th International Leksell Gamma Knife Society Meeting. Amsterdam: Leksell Gamma Knife Society; 2015.

